# Surgically Managed Perforated Jejunal Diverticulitis

**DOI:** 10.7759/cureus.15930

**Published:** 2021-06-25

**Authors:** Vlad Vayzband, Hamza Ashraf, Paola Esparragoza

**Affiliations:** 1 Internal Medicine, Saint Peter’s University Hospital, New Brunswick, USA; 2 Gastroenterology and Hepatology, Saint Peter’s University Hospital, New Brunswick, USA

**Keywords:** jejunal diverticulitis, jejunal perforation, small bowel diverticulosis, diverticular disease, dysmotility, intraluminal pressure, peritonitis

## Abstract

A 71-year-old male with a past medical history significant for chronic constipation presented to the emergency department for acute onset of severe abdominal pain. On presentation, the patient appeared to be in distress, exemplifying signs of peritonitis despite vital signs being grossly benign. CT scan established the diagnosis of a perforated jejunal diverticulitis. Initially, the patient was managed conservatively with IV fluids, antibiotics, and pain control medications. Diagnostic imaging in tandem with the patient's failure to improve incited surgical intervention with a jejunal resection and establishment of a primary anastomosis. This case illustrates additional differential diagnoses necessary for consideration in an elderly patient presenting with an acute abdomen.

## Introduction

Diverticulosis is a condition in which several pocket-like outpouchings develop within the wall of the gastrointestinal track as a result of herniations of the mucosa and submucosa through defects in the muscular propria [1,2]. The pathogenesis of diverticula formation, initially attributed to environmental factors such as diet, is now considered to be multifactorial and has links to both environmental and genetic components [3]. One of the more common sequelae of diverticulosis is inflammation and infection of the individual outpouchings, also known as diverticulitis. Consequently, diverticulitis can lead to serious complications ranging from abscess formation and bowel obstruction to bowel perforation and peritonitis [4]. Diverticula are most commonly found in the colon, specifically at the level of the sigmoid colon, at points weakened by the penetration of blood vessels known as the vasa recta [1,3,5]. Diverticular disease exclusive of Meckel’s diverticulum is significantly less common in the small intestine as compared to the large intestine [6]. Herein, we describe a rare case of jejunal diverticulitis complicated by perforation and peritonitis that was managed surgically through small bowel resection.

## Case presentation

**Figure 1:**
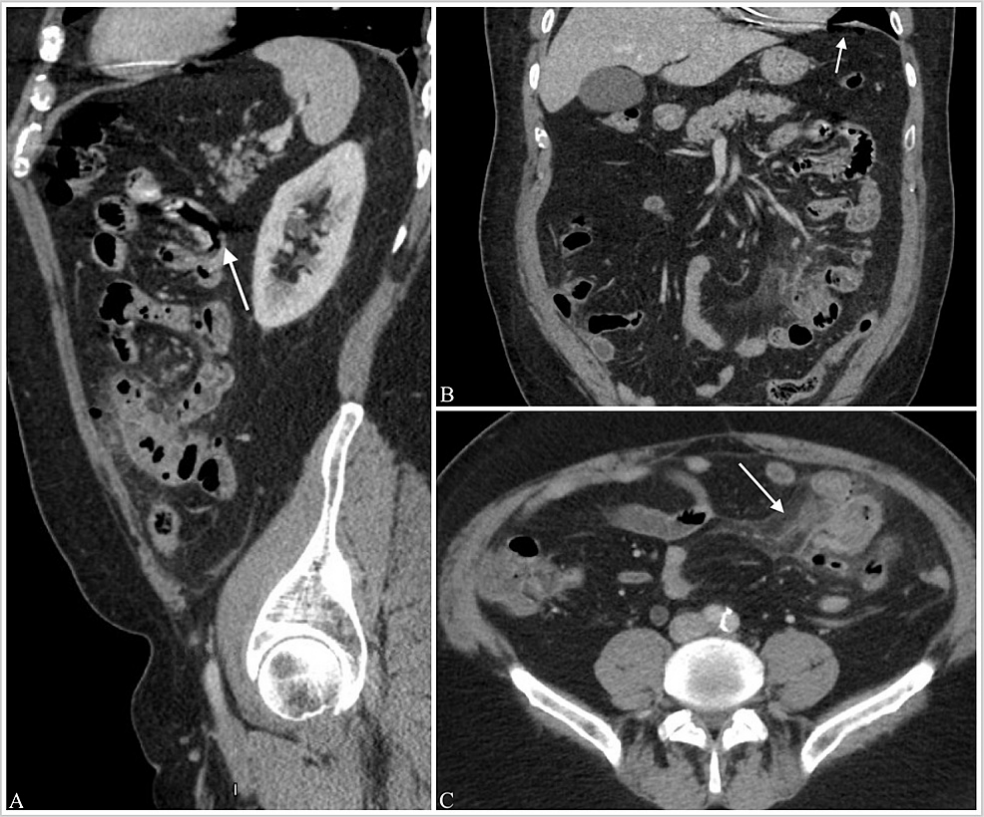
IV contrast CT scans showing (A) area of perforation, (B) sub-diaphragmatic free air, (C) pericolic fat stranding with inflammation and edema

A 71-year-old Caucasian male with a past medical history of chronic constipation, depression, hypertension, and hypothyroidism presented to the emergency department complaining of severe abdominal pain that began suddenly the day prior. The pain, described as being constant, sharp, and “crampy,” was initially localized to the left upper quadrant but had progressed to involve the entire abdomen just prior to presentation. The patient also complained of fevers and rigors throughout the day, with a sublingual temperature check at home showing 100.5 degrees Fahrenheit, leading the patient to start acetaminophen. There was no reporting of nausea, vomiting, or diarrhea. Vitals on presentation included a temperature of 98.1 degrees Fahrenheit, a heart rate of 91 beats per minute, a blood pressure of 140/86 mmHg, a respiratory rate of 16 breaths per minute, and an oxygen saturation of 99% on room air. Physical examination revealed diffuse abdominal tenderness that was worst at the upper left quadrant, guarding of the entire abdomen, and rebound tenderness. Laboratory tests were significant for an elevated white blood cell count of 22.6 x 10⁹/L with a neutrophil predominance (18.7 x 10⁹/L). The levels of lipase and lactic acid were found to be within normal limits, with readings of 13 U/L and 1.0 mmol/L, respectively. Other laboratory values were also found to be grossly normal. A CT scan of the abdomen and pelvis with IV contrast disclosed multiple foci of regional and upper abdominal peritoneal free air secondary to perforated jejunal diverticulitis without a drainable fluid collection (Figure 1). The patient was initially started on IV fluids, IV acetaminophen, and IV piperacillin-tazobactam. After the results of the CT scan came back, the patient was taken to the operating room for an exploratory laparotomy, which showed approximately 75 cm of jejunum, which was tan-brown and hemorrhagic with areas of suppurative yellow material. The affected area was then excised with establishment on a primary anastomosis. Intraluminal examination of the resected bowel showed areas of induration with multiple diverticular tracts impacted with fecal material. The perioperative course was unremarkable with the remainder of the patient’s hospital stay consisting of an uncomplicated two days of observation.

 

## Discussion

Diverticular disease of the colon is a relatively common disorder. Its pathogenesis is nested within a multifactorial etiology with risk factors including a high-fat diet, large consumption of red meat, low fiber, physical inactivity, and increasing age [3]. Genetic predispositions surrounding structural protein defects and neurologic impairments have also been implicated but to a lesser degree [7,8]. The summation of all these potential risk factors focalizes upon the idea of a structural defect at a precise anatomic location. Predisposing factors for diverticula formation occur at anatomical weak points contained within the muscularis propria, irrespective of the location within the gastrointestinal tract. Extraluminal duct and vascular entrance, along with intrinsic weakness to the muscular layer, ultimately culminate in mucosal and submucosal protrusion often being described as a pseudodiverticula [9]. Contributory processes that include visceral vasculopathies, myopathies, and neuropathies further elucidate the medium of its pathogenesis. Despite this, the exact mechanism for jejunal diverticula formation has not been established. The current proposed mechanism consists of abnormal intestinal peristalsis, which leads to increased intraluminal pressures with outpouching occurring at potential points of weakness [10]. Areas at which the blood supply enters offer a potential area for diverticula formation and are most often seen in the proximal segments of the jejunum. Distinct from its distal portion, the vascular supply configures into simple arcades with long and relatively fewer vasa recta. Reduction of blood supply by means of luminal narrowing confers potential risk to muscular layer atrophy. This may offer an explanation to the peak incidence rates of jejunal diverticula being within the sixth to seventh decade with an overall male predominance, which seemingly correlates with atherosclerotic risk factors and vascular senescence [11-13]. Its disruption further manifests as atrophy to muscular and neurological components contained within the muscularis propria. This disruption may then lead to intestinal dyskinesis generating slower transit rates, increased intraluminal pressure, and eventual protrusion through the area of weakened muscle. Therefore, any risk factor that further slows down peristalsis or increases the density of the chyme will also exacerbate the rate at which diverticula form. Similar conclusions were also drawn in a study that analyzed the involvement of the small intestine in systemic sclerosis. Vascular ischemia with smooth muscle atrophy and subsequent fibrosis were determined to be the crucial components that manifested as impaired motility [14]. Likewise, neurological impairments that are seen in Fabry’s disease also manifested as intestinal dysmotility and increased intraluminal pressures [15]. As a pathology that involves multiple different systems it becomes difficult to precisely articulate which is the initiator, yet despite this, a commonality among all remains consistent, an enteric neuropathy. Back in 1965, Macbeth and Hawthorn first described diverticular disease as an acquired neuromuscular derangement [16]. Since then, morphometric analysis of the enteric nervous system showed significant reduction of neuronal density in all enteric nervous plexuses in patients suffering from diverticular disease [17]. For this reason the clinical features are often non-specific, encompassing constipation, abdominal discomfort, flatulence, malnutrition, and dyspepsia. Due to a lack of acute and severe features, patients present late in the disease course, often when complications arise. Notably the most common complications are perforation, intestinal obstruction, and diverticular bleeding [18]. In a similar fashion to that of the Hinchey classification of colonic diverticulitis, jejunal complication can follow a similar progression. With continued inflammation of the diverticula, abscess formation can occur with subsequent extension into the abdomen and liver, with potential to perforate and cause peritonitis [19]. As the presentation of an acute abdomen yields various differential diagnoses, suspicion for a small intestine perforation may be arrived upon by first excluding other more prominent causes. Endoscope and other commonly used imaging modalities including ultrasound and plain radiographs have not been able to show the prominent features necessary for diagnosis [20]. However, various case studies that dealt with jejunal diverticulitis have shown that CT scans with oral and rectal contrast were able to localize areas of disease, showing features of enhanced wall thickness, air extravasation, and inflammatory changes to surrounding areas [20]. Currently, the management of jejunal diverticulitis has not been established, with treatment strategies primarily centered around the patient's symptoms. Mild inflammation with no peritoneal signs can be managed conservatively with bowel rest, broad-spectrum antibiotics, and hydration. In cases in which abscess formation or intraperitoneal collections arise, CT-guided aspiration and drainage can be performed [21]. When conservative measures fail or peritoneal signs become present, prompt segmental resection with a primary anastomosis becomes necessary [22].

## Conclusions

Jejunal diverticulitis is a rare but a potentially fatal differential diagnosis for an acute abdomen. Elderly patients commonly manifest acute symptoms late in the disease course. As such, an acute presentation of left upper quadrant pain, under non-emergent conditions, may warrant an imaging modality, which is not commonly used for routine diagnosis. As many of the common imaging modalities yield inconclusive results, high clinical suspicions must be undertaken. CT with oral and IV contrast has been shown to be the imaging modality most useful in excluding other diseases of the gastrointestinal tract and in establishing a targeted diagnosis, with pharmacological and surgical intervention being guided by the patient's presenting symptoms.
